# Ideal Kerker scattering by homogeneous spheres: the role of gain or loss

**DOI:** 10.3762/bjnano.13.73

**Published:** 2022-08-24

**Authors:** Qingdong Yang, Weijin Chen, Yuntian Chen, Wei Liu

**Affiliations:** 1 School of Optical and Electronic Information, Huazhong University of Science and Technology, Wuhan, Hubei 430074, P. R. China; 2 Wuhan National Laboratory for Optoelectronics, Huazhong University of Science and Technology, Wuhan, Hubei 430074, P. R. China; 3 College for Advanced Interdisciplinary Studies, National University of Defense Technology, Changsha, Hunan 410073, P. R. China

**Keywords:** gain and loss, Kerker scattering, Mie particle

## Abstract

We investigate how the optical gain or loss (characterized by isotropic complex refractive indexes) influence the ideal Kerker scattering of exactly zero backward scattering. It was previously shown that, for non-magnetic homogeneous spheres with incident plane waves, either gain or loss prohibit ideal Kerker scattering, provided that only electric and magnetic multipoles of a specific order are present and contributions from other multipoles can all be made precisely zero. Here we reveal that, when two multipoles of a fixed order are perfectly matched in terms of both phase and magnitude, multipoles of at least the next two orders cannot possibly be tuned to be all precisely zero or even perfectly matched, and consequently cannot directly produce ideal Kerker scattering. Moreover, we further demonstrate that, when multipoles of different orders are simultaneously taken into consideration, loss or gain can serve as helpful rather than harmful contributing factors, for the elimination of backward scattering.

## Introduction

The original Kerker scattering of zero backward scattering was first proposed for homogeneous magnetic spheres with equal electric permittivity and magnetic permeability ε = μ [1]. This proposal had not attracted much attention for a long time, mainly due to the scarcity of magnetic materials, especially at the high-frequency spectral regimes. In the past decade, thanks to the explosive developments of metamaterials and metasurfaces, the underlying core concept of optically induced magnetism in non-magnetic structures has invigorated and completely transformed Kerker’s original proposal (see the reviews [2–4]). The fusion of optically induced magnetism with Kerker scattering by high-index materials [5] has rendered new perspectives for photonic studies concerning not only scattering of individual particles or their finite clusters [6–7], but also of extended periodic or aperiodic structures [2–4,8–14]. Moreover, this significantly broadened concept of Kerker scattering has rapidly penetrated into other disciplines of photonics, revealing hidden connections between seemingly unrelated concepts and demonstrations [15–23].

In the original proposal for homogenous spheres with ε = μ, electric and magnetic multipoles of all orders are automatically perfectly matched in terms of both phase and magnitude [24], leading to ideal Kerker scattering of exactly zero backward scattering [1]. Nevertheless, for demonstrations relying on optically induced magnetism with non-magnetic structures (μ = 1), it is rather challenging, if not impossible, to precisely match all multipoles simultaneously, ending up with only significantly suppressed but not exactly zero backward scattering [2–4]. Quite recently, Olmos-Trigo et al. revisited the simplest case of a non-magnetic isotropic and homogeneous sphere with incident plane waves and concluded that: (i) ideal zero backward scattering is achievable only for materials without gain or loss (characterized by real refractive indexes) [25], and (ii) extra gain or loss inhibit such ideal Kerker scattering. Besides the proven feasible perfect matching of electric and magnetic multipoles of one specific fixed order, the validity of the conclusion resides on the additional assumption that magnitudes of multipoles of all other orders can be simultaneously tuned to be perfectly zero. For general discussions of optical properties, such as scattering and absorption cross sections, it is physically legitimate to take into consideration only those dominant contributing multipole terms and drop other minor ones (such as in the widely adopted dipole approximation). While for the investigation into the extreme case of ideal zero backward scattering, those minor multipole terms cannot be simply discarded unless they are exactly zero or also perfectly matched in a similar fashion.

In this work we show that, despite the previously proven fact that multipoles of a fixed order can be perfectly matched in the absence of loss or gain [25], the contributions from multipoles of at least the next two orders cannot be simultaneously tuned to be all zero or perfectly matched. In other words, ideal Kerker scattering of exact zero backward scattering is not directly achievable through matching a pair of multipoles of one specific order only. We further reveal that when multipoles of different orders are all taken into consideration, loss or gain should be employed rather than avoided for the elimination of backward scattering. It is shown that, at the presence of multipoles of various orders, the absence of backward scattering can be obtained through tuning the refractive index on the complex plane, breaking the connection between zero backscattering and helicity conservation.

## Results

### Formulas and analysis of ideal Kerker scattering

For the scattering of incident linearly polarized plane waves (wavelength λ and angular wavenumber *k* = 2π/λ) by homogeneous non-magnetic spheres (isotropic refractive index *m*, radius *R*, and normalized geometric parameter *x* = *kR*), the scattered fields can be expanded into a series of electric and magnetic multipoles of order *l* (*l* = 1 corresponds to dipoles). They are characterized, respectively, by the complex Mie coefficients *a_l_* and *b_l_* [26–27]:


[1]

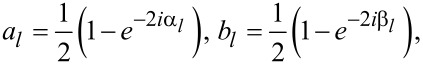



where α*_l_* and β*_l_* are complex phase angles (they are real when *m* is real). Those phase angles can be obtained through the following relations [26]:


[2] $$\begin{array}{c} \tan\alpha_{l}=-\frac{S_{l}^{\prime }\left(mx\right)S_{l}\left(x\right)-mS_{l}\left(mx\right)S_{l}^{\prime }\left(x\right)}{S_{l}^{\prime }\left(mx\right)C_{l}\left(x\right)-mS_{l}\left(mx\right)C_{l}^{\prime }\left(x\right)},\\ \tan\beta_{l}=-\frac{mS_{l}^{\prime }\left(mx\right)S_{l}\left(x\right)-S_{l}\left(mx\right)S_{l}^{\prime }\left(x\right)}{mS_{l}^{\prime }\left(mx\right)C_{l}\left(x\right)-S_{l}\left(mx\right)C_{l}^{\prime }\left(x\right)}. \end{array}$$

Here the prime ′ denotes first-order derivative with respect to the entire argument in the bracket; *S_l_*(*z*) = *zj_l_*(*z*) and *C_l_*(*z*) = −*zy_l_*(*z*) are Riccati–Bessel functions; *j_l_*(*z*) and *y_l_*(*z*) are spherical Bessel functions of the first and second kinds.

With *a_l_* and *b_l_* obtained, the total scattering efficiency can be calculated through [26–27]:


[3]

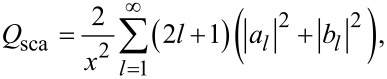



and the ideal Kerker scattering in terms of backward scattering efficiency *Q*_b_ can be expressed as [26–27]:


[4] $$Q_{\text{b}}=\frac{1}{x^{2}}{\left|\sum_{l=1}^{\infty }\left(2l+1\right){\left(-1\right)}^{l}\left(a_{l}-b_{l}\right)\right|}^{2}=0.$$

Equation 4 has an infinite set of possible solutions, and what is discussed [25] is actually the following very special scenario:


[5] $$a_{l}=b_{l}\neq 0,\;l=l_{0},$$

[6] $$a_{l}=b_{l}=0,\;l\neq l_{0},$$

where *l*_0_ is an arbitrary natural number and a pair of multipoles of order *l*_0_ are perfectly matched as shown in Equation 5. The significant contribution from [25] is to prove rigorously that Equation 5 has a solution only when *m* is real, meaning that, at the presence of loss or gain, multipoles of the same order cannot be ideally matched. Despite this seminal contribution, there is a problem that in [25] it has not been discussed whether Equation 5 and Equation 6 are really compatible. Such discussions concerning compatibility are vitally important, since Equation 5 will not necessarily lead to ideal Kerker scattering of precise zero backscattering.

### Mismatch among multipoles of three successive orders

In this section, we aim to prove that Equation 5 and Equation 6 are not exactly compatible, thus proving that Ideal Kerker scattering of exact zero backward scattering is actually inaccessible through matching multipoles of a specific order only. For all our following discussions, the obviously trivial scenario of *m* = 1 (we assume that the background medium is air of index 1 throughout our study) or *R* = 0 is excluded. For another special case of zero index *m* = 0, the Mie coefficients can be simplified as (as *m*→0) [26–27]:


[7] $$a_{l}=\frac{S_{l}\left(x\right)}{T_{l}\left(x\right)},\;\;b_{l}=\frac{S_{l}^{\prime }\left(x\right)S_{l}\left(mx\right)}{T_{l}^{\prime }\left(x\right)S_{l}\left(mx\right)},$$

where 
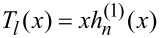
, and 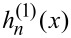 is a spherical Hankel function of the first kind. Since *S_l_*(*mx*)→0 when *m*→0, we get a definite *a_l_* but indefinite *b_l_* (L’Hôpital’s rule will not help to make *b_l_* definite, since the zero term in the numerator and denominator is the same [28]). So it has been proved that for *m* = 0, there are no definite scattering properties for ideally monochromatic plane waves. Physical investigations can be implemented only after considering simultaneously the dispersion of the index and the spectrum of the incident waves. Consequently, the zero-index scenario is also excluded in the following analysis.

It has been rigorously proved that the solutions of Equation 5 satisfy either of the following equations:


[8] $$S_{l_{0}}\left(mx\right)=0,$$

[9] $$S_{l_{0}}^{\prime }\left(mx\right)=0,$$

which do not have a common solution according to the Brauer–Siegel theorem [29–30]. Similarly, to prove that then multipoles of all other orders (*l* ≠ *l*_0_) cannot all be perfectly matched (of which that other multipoles cannot be tuned to be all zero is merely a special scenario), it is more than sufficient to prove that there exists one multipole order *l*_1_ (*l*_1_ ≠ *l*_0_) for which:


[10] $$S_{l_{1}}\left(mx\right)\cdot S_{l_{1}}^{\prime }\left(mx\right)\neq 0.$$

Obviously, Equation 10 ensures that 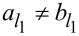, meaning that Equation 6 cannot be simultaneously met.

According to the following recurrence relations of Riccati–Bessel functions [29]:


[11] $$S_{l_{0}+1}^{\prime }\left(mx\right)=-\frac{l_{0}+1}{mx}S_{l_{0}+1}\left(mx\right)+S_{l_{0}}\left(mx\right),$$

[12] $$S_{l_{0}}^{\prime }\left(mx\right)=\frac{l_{0}+1}{mx}S_{l_{0}}\left(mx\right)-S_{l_{0}+1}\left(mx\right),$$

(i) when 
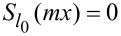
 and 
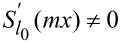
: According to Equation 12, we obtain 
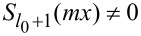
. This together with Equation 11 leads to 
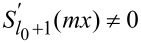
. As a result, Equation 10 is satisfied at least for *l*_1_ = *l*_0_ + 1, securing that 
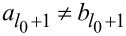
.

(ii) When 
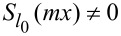
 and 
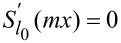
: Also, according to Equation 12, we get 
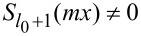
. Nevertheless, according to Equation 11, 
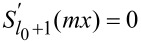
 if the following conditions can be met:


[13] $$S_{l_{0}+1}\left(mx\right)=\pm S_{l_{0}}\left(mx\right),\;l_{0}+1=\pm mx.$$

Nevertheless, following the same logic, extending the multipole matching to the next order *l*_0_ + 2 requires:


[14] $$S_{l_{0}+2}\left(mx\right)=\pm S_{l_{0}+1}\left(mx\right),\;l_{0}+2=\pm mx.$$

It is quite obvious that Equation 13 and Equation 14 can not be simultaneously satisfied, that is, *mx* cannot be both ±(*l*_0_ + 1) and ±(*l*_0_ + 2), and, thus, multipole mismatch happens at least for *l*_1_ = *l*_0_ + 2: 
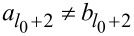
.

The arguments above, consistent with a recent study [30], confirm that when a multipole of a specific order *l*_0_ is perfectly matched in a nontrivial way, Equation 5, the scattering contributions from multipoles of at least the next two successive orders (*l*_0_ + 1 and *l*_0_ + 2) cannot be simultaneously tuned to be zero or matched. In other words, perfect matching of multipoles of one specific order does not guarantee ideal zero backward scattering.

### Effects of gain or loss on ideal Kerker scattering: non-resonant regimes

**Figure 1:**
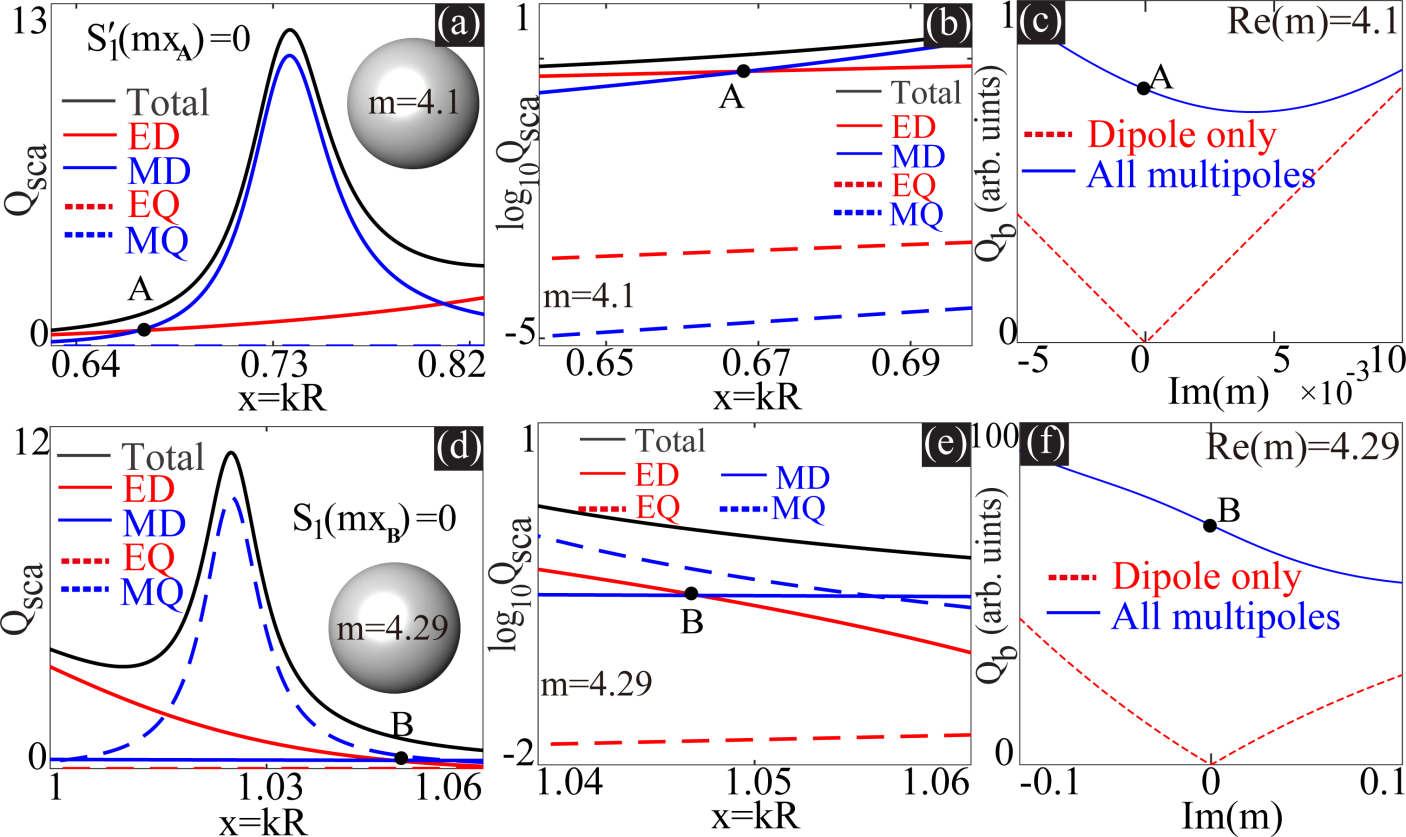
Scattering spectra (both total scattering and the contributions from different multipoles are included) are shown in (a) and (b) for *m* = 4.1, and in (d) and (e) for *m* = 4.29. Here, (b) and (e) are sections of (a) and (b), respectively, which are close to the dipole matching points and enlarged for clarity. For each scenario, there is a indicated point (*x***_A_** ≈ 0.6684 and *x***_B_** ≈ 1.0472) where the dipoles are perfectly matched. (c, f) Dependence of *Q*_b_ on Im(*m*), at *x***_A_** with fixed Re(*m*) = 4.1 and at *x***_B_** with fixed Re(*m*) = 4.29, respectively. In (c) and (f), two sets of results are shown, considering only dipoles or all multipoles, respectively.

We show in Figure 1 two scenarios where the electric and magnetic dipoles (ED and MD) are perfectly matched in non-resonant spectra regimes. The scattering efficiency spectra (scattering efficiency *Q*_sca_ as a function of *x* = *kR*) for a homogeneous sphere (*m* = 4.1) are shown in Figure 1a, where both total scattering and the contributions from different multipoles (dipoles and electric and magnetic quadrupoles: EQ and MQ) are included. This is actually the case studied in detail in [25]. The ED and MD are perfectly matched at *x***_A_** = 0.6684, where 
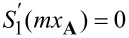
. As argued in the last section, at *x***_A_**, scattering from multipoles of higher orders is not exactly zero (see Figure 1b, which shows an enlarged part of the spectra close to *x***_A_** in logarithmic scale), though they are much smaller than those of dipoles. For explorations of general properties like scattering and absorption cross sections, it is fine to drop those quadrupole terms and to keep the dipole terms only. Nevertheless, for the study of the extreme case of ideal Kerker scattering, simply discarding those higher-order terms cannot be justified and could even lead to inaccurate conclusions.

To verify the claim above, we show in Figure 1c the dependence of the backward scattering efficiency *Q*_b_ at *x***_A_** on the imaginary part of refractive index Im(*m*); the real part of *m* is fixed at Re(*m*) = 4.1. Im(*m*) *>* 0 and Im(*m*) *<* 0 correspond to loss and gain, respectively. Here two sets of spectra are shown, for which either only dipoles or multipoles of all orders are taken into consideration. It is clear from Figure 1c that, when only dipoles are considered, ideal Kerker scattering is achieved when *m* is real, and any extra loss or gain would inhibit such scattering, as is the major conclusion of [25]. In sharp contrast, when all multipoles are considered, ideal Kerker scattering is not accessible at the perfect matching point of dipoles anymore. Moreover, as shown in Figure 1c, extra loss can be employed to further suppress the backward scattering, serving as a friend rather than a foe for the Kerker scattering. Another scenario of perfect dipole matching at *x***_B_** = 1.0472 for *m* = 4.29 is summarized in Figure 1d–f, for which the other perfect matching condition is satisfied, that is *S*_1_(*mx***_A_**) = 0. Here the effects of higher-order multipoles are even more pronounced (see Figure 1f) since the magnitudes of dipoles and higher multipoles are comparable (see Figure 1e.

### Effects of gain or loss on ideal Kerker scattering: resonant regimes

**Figure 2:**
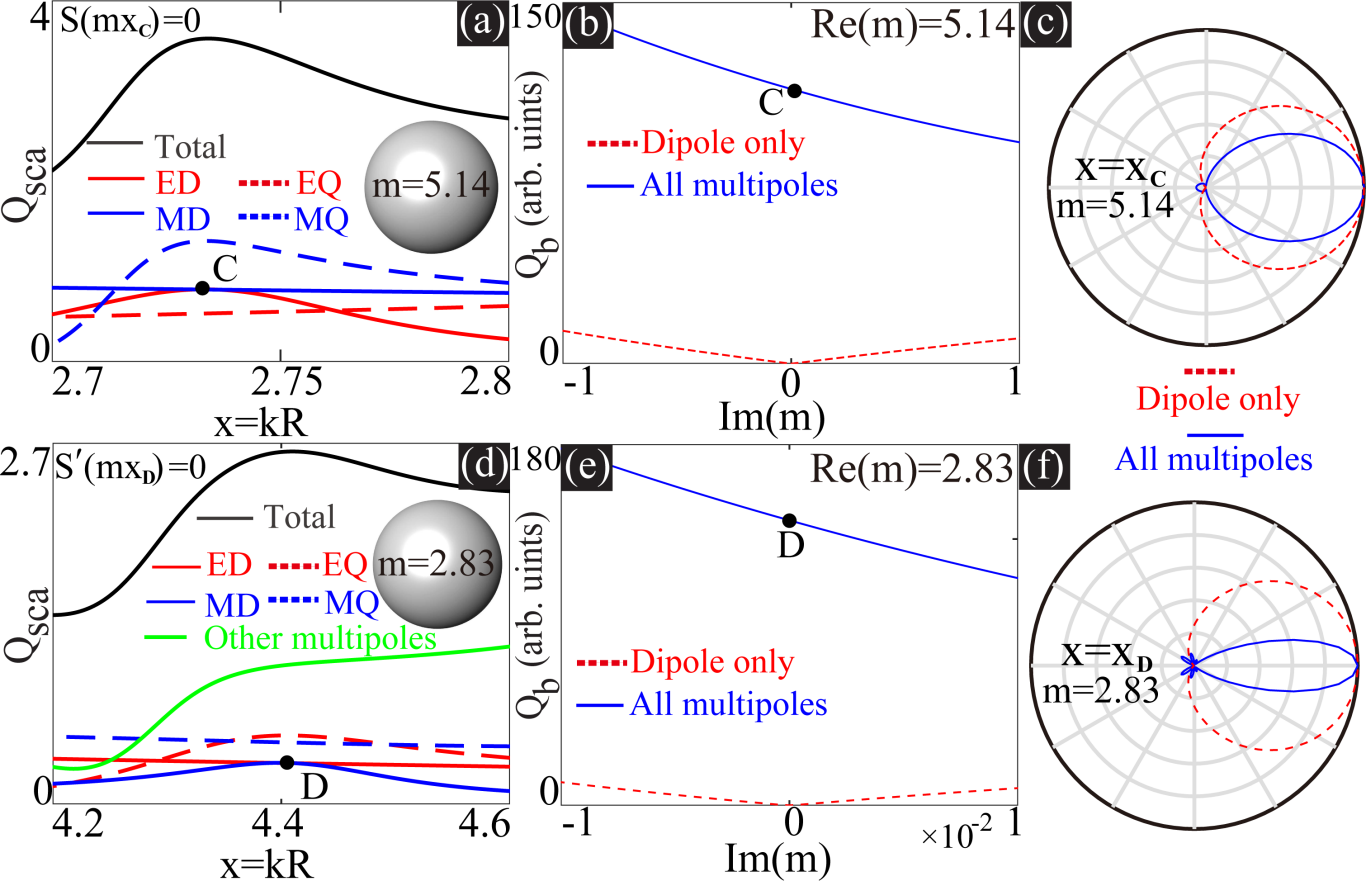
Scattering spectra are shown in (a) for *m* = 5.14 and in (d) for *m* = 2.83. For each scenario, there is an indicated point (*x***_C_** ≈ 2.7366 and *x***_D_** ≈ 4.4123) where the dipoles are perfectly matched. (b, e) Dependence of *Q*_b_ on Im(*m*) at *x***_C_** with fixed Re(*m*) = 5.14 and at *x***_D_** with fixed Re(*m*) = 2.83, respectively. (c, f) The 2D angular scattering patterns (in the plane parallel to both polarization and incident directions) at *x***_C_** with *m* = 5.14 and at *x***_D_** with *m* = 2.83, respectively. In (b, c) and (e, f) two sets of results are shown, considering only dipoles or all multipoles, respectively.

In the last section, we discussed only the perfect dipole matching at the non-resonant regimes, where not only the backward scattering is suppressed, but also the overall scattering is small. Such scattering is of very limited significance, since what is widely required in photonics is suppressed backward scattering accompanied by large total scattering [2–4]. In this section, we move to the resonant regimes where the dipoles can be perfectly matched. Two such scenarios are summarized in Figure 2, where the conditions of *S*_1_(*mx***_C_**) = 0 and 
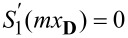
 are satisfied, in Figure 2a–c with *x***_C_** ≈ 2.7366, *m* = 5.14, and in Figure 2d–f with *x***_D_** ≈ 4.4123, and *m* = 2.83), respectively. In Figure 2, besides the scattering spectra (Figure 2a,d) and dependence of *Q*_b_ on Im(*m*) (Figure 2b,e), we show also the two-dimensional (2D) scattering patterns (in the plane parallel to both the incident and polarization directions of the independent plane waves) at the dipole matching points (Figure 2c,f).

As indicated by the scattering spectra, the scattering by the higher-order multipoles is rather strong, which ruins the ideal Kerker scattering (see Figure 2b,e) and makes the overall patterns considering all multipoles (solid lines of Figure 2c,f) contrastingly different from those of matched dipoles only (dashed lines of Figure 2c,f). Similar to what is shown in Figure 1, when all multipoles are considered, extra loss can be employed to further suppress the backward scattering, serving as a constructive rather than a destructive factor for demonstrations of Kerker scattering.

### Kerker scattering without multipole matching of any specific order

**Figure 3:**
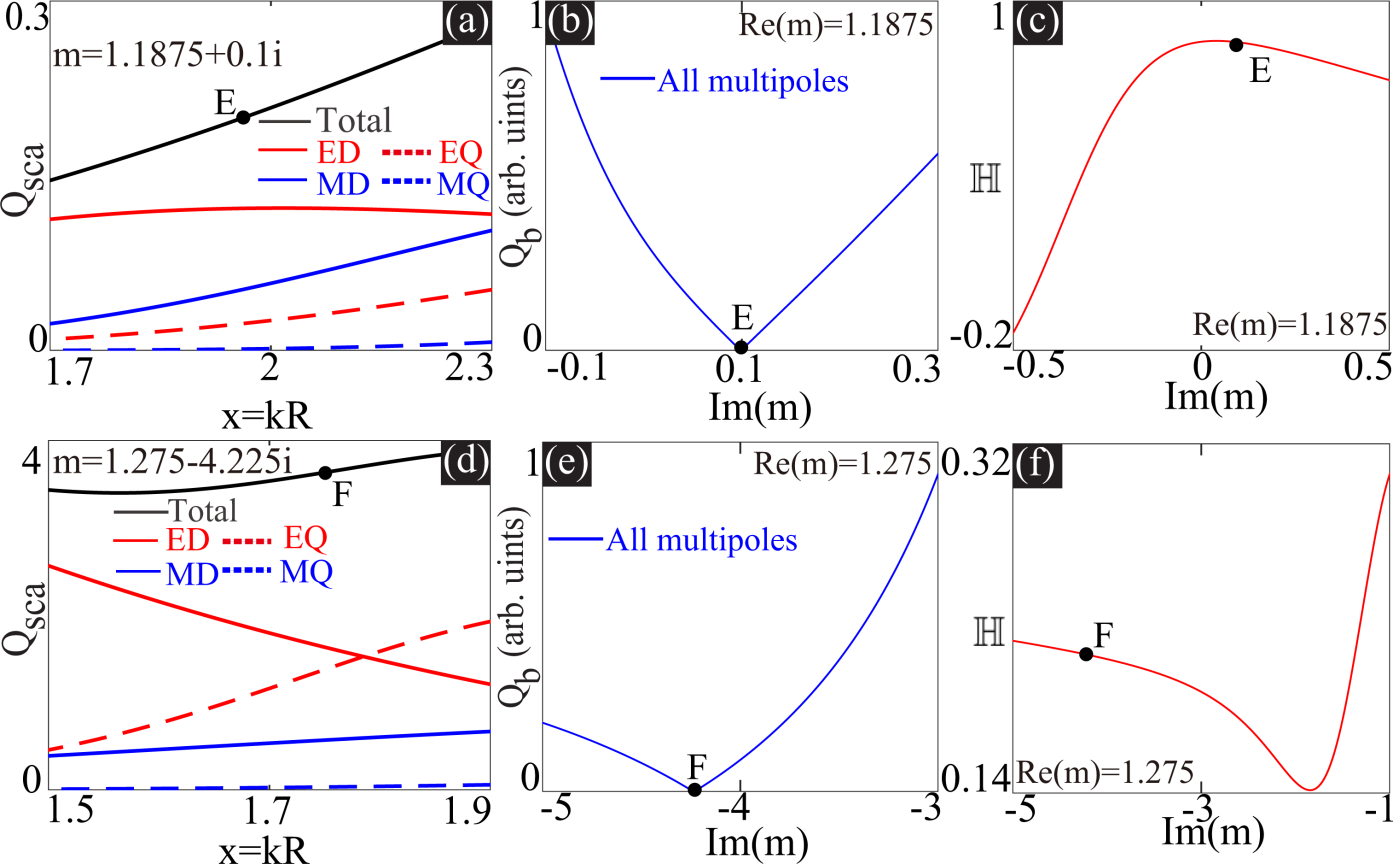
Scattering spectra are shown in (a) for *m* = 1.1875 + 0.1*i* (with loss) and in (d) for *m* = 1.275 − 4.225*i* (with gain). For each scenario, there is an indicated point (*x***_E_** ≈ 1.9591 and *x***_F_** ≈ 1.7492) where the backward scattering is eliminated, as confirmed in (b) and (e). Dependence of *Q*_b_ (b, e) and 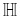 (c, f) on Im(*m*), at *x***_E_** with fixed Re(*m*) = 1.1875 and at *x***_F_** with fixed Re(*m*) = 1.275.

We have confirmed in the last sections, by both mathematical analysis and numerical calculations, that perfect matching of multipoles of a specific order does not necessarily produce ideal Kerker scattering due to non-negligible higher-order multipoles. Moreover, those higher-order terms would make the extra gain or loss a constructive factor for further suppression of the backward scattering. Now we come back to Equation 4, the solution of which does not really require multipole matching of any specific order (such as those shown in Equation 5 and Equation 6), but can be obtained through fully destructive interferences among multipoles of several orders along the backward direction. Such an effect is also termed as “generalized Kerker effect”, originating from interferences among multipoles of different orders [4,31–34]. Generally speaking, to obtain zero backward scattering with complete destructive interferences among multipoles, at least two multipoles of opposite parities are needed. This could be a pair of multipoles of the same order (such as ED and MD), or two multipoles of different orders (such as ED and EQ, or MD and MQ), or more than two multipoles that are not of the same parity [4,31]. For further confirmation, we show two such scenarios with loss or gain in Figure 3, where Kerker scattering is observed, in Figure 3a,b (*x***_E_** ≈ 1.9591, *m* = 1.1875 + 0.1*i*), and in Figure 3d,e (*x***_F_** ≈ 1.7492 for *m* = 1.275 − 4.225*i*), respectively. Figure 3a,b shows that there is no non-trivial perfect multipole matching (*a_l_* = *b_l_* ≠ 0) at the indicated positions, despite which the Kerker scattering can still be achieved (see Figure 3b,e at *x***_E_** and *x***_F_**). Moreover, the dependence of *Q*_b_ on Im(*m*) (Figure 3b,e) can confirm that the selected loss or gain is vitally important for such achievement, as a little detuning from them would immediately ruin the Kerker scattering. For both scenarios, it is quite obvious that to fix the index to be real is actually harmful for the suppression of backward scattering.

It has been rigorously proved that *n*-fold (*n* ≥ 3) rotation symmetry together with helicity conservation would automatically guarantee ideal Kerker scattering of zero backward scattering [22,35]. For homogenous sphere scattering with incident plane waves, the rotation symmetry is secured (*n* = ∞) and the helicity conservation requires the multipole matching of all orders. Consequently, Kerker scattering obtained through perfect matching of multipoles at each order are inextricably connected through helicity conservation, as is confirmed in [25]. Nevertheless, we have shown in the last section that Kerker scattering is also achievable without multipole matching of any specific order, for which it is expected that the connection between Kerker scattering and helicity conservation would be broken. To confirm this, we further show the dependence of the helicity conservation factor 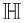 on Im(*m*) in Figure 3c,f. Here 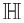 is defined as [25,36]:


[15]

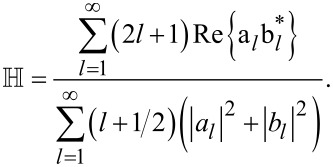



Here 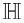 = 1 corresponds to ideal helicity conservation, which means that for incident circularly polarized plane waves, the waves scattered along all directions are also circularly polarized of the same handedness (including the special case of zero scattering) [17,22,37–38]. A comparison between Figure 3c,f and Figure 3b,e can confirm that there is no connection between the Kerker scattering and helicity conservation, since 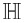 is far from unity at the indicated Kerker scattering points (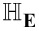 = 0.857, 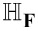 = 0.2115). In other words, rotation symmetry and helicity conservation lead to zero backward scattering, while rotation symmetry and zero backward scattering does not necessarily imply helicity conservation.

## Discussion

There are several significant points worth emphasizing at the end: (i) For numerical demonstrations of perfect multipole matching, we discuss only dipoles while the principles revealed are applicable for multipoles of any order. (ii) In this study, we only discuss Kerker scattering of zero backward scattering (first Kerker scattering). For the second Kerker scattering of zero forward scattering, despite the inevitable involvement of gain materials as required by optical theorem, multipoles of various order rather than a specific order should be taken into considerations simultaneously, as has been implemented in this work. (iii) Is ideal Kerker scattering of exact zero backward scattering achievable, in a rigorously mathematical sense, with homogenous non-magnetic spheres? The answer is: We do not know. It is well known that for arbitrary algebraic equations of order *L*,


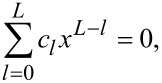


for which *L* is a finite natural number and *c_l_* are complex constant coefficients, the fundamental theorem of algebra secures that there is at least one solution on the complex *x*-plane [28]. Nevertheless, Equation 4 is a transcendental rather than an algebraic equation, of which the existence of exact solution on the complex plane is not definite. Such a transcendental equation can be only tackled through numerical analysis and, thus, numerical errors make it impossible to decide if the Kerker scattering demonstrated in Figure 3 is ideal or not in a mathematical sense. (iv) If an exact solution of Equation 4 exists, the chances of this solution being complex are much higher than it being purely real (real axes cover a tiny part of the complex plane). If an exact solution does not exist, the backward scattering is minimized more probably at complex arguments rather than at purely real ones. As a result, gain or loss are definitely helpful rather than harmful for the realizations of ideal Kerker scattering or suppression of backward scattering. (v) Discussing the exact solution of Equation 4 (and thus ideal Kerker scattering) is interesting and meaningful only mathematically. From a physical perspective, such an exploration is of very little significance, if not of no significance at all. This is because for realistic observations, there is no absolute boundary between exactly zero and approximately zero, which highly depends on the resolutions of different equipments. Moreover, when the scattering intensity gets smaller and smaller, the optical regime we study will shift from wave optics to quantum optics, where the quantum fluctuations would play a non-negligible role [39]. Then wave optics and, thus, Equation 4 itself breaks down and it becomes meaningless to discuss its exact solution.

## Conclusion

We have proved that perfectly matching electric and magnetic multipoles of a specific order do not necessarily produce ideal Kerker scattering of exact zero backward scattering. This is because no matter how small the contributions from other multipoles are, they can never be made to be all zero or perfectly matched. In other words, to obtain zero backward scattering, we cannot just consider multipoles of a specific order. Instead we need to consider all contributing ones that are not exactly zero. It is further demonstrated that when multipoles of various order are simultaneously considered, loss or gain can be employed for suppression of backward scattering, serving as beneficial rather than detrimental contributions for the realization of ideal Kerker scattering. When Kerker scattering is achieved through the destructive interference among multipoles of several orders in the backward direction, rather than perfect multipole matching of each order, it is not synonymous with helicity conservation any more.
